# Students' Motivation for Sport Activity and Participation in University Sports: A Mixed-Methods Study

**DOI:** 10.1155/2018/9524861

**Published:** 2018-06-12

**Authors:** Katharina Diehl, Anna Katharina Fuchs, Katharina Rathmann, Jennifer Hilger-Kolb

**Affiliations:** ^1^Mannheim Institute of Public Health, Social and Preventive Medicine, Medical Faculty Mannheim, Heidelberg University, Ludolf-Krehl-Straße 7-11, 68167 Mannheim, Germany; ^2^Faculty of Rehabilitation Sciences, Technical University Dortmund, Emil-Figge-Straße 50, 44227 Dortmund, Germany

## Abstract

**Background:**

Physical activity among students is essential for complimenting sedentary behavior and for individuals' future health. This study investigates reasons for sport engagement among students and addresses the utilization of university sports programs (USP) by employing a mixed-methods approach.

**Methods:**

The NuPhA-Study consists of a quantitative online survey (n=689) followed by qualitative interviews (n=20). In the survey, we assessed reasons for sport activity using a 24-item battery and USP utilization. Quantitative results were further explored using qualitative data to check for completeness of the predefined items (content validity) and to identify opportunities to improve participating in USP.

**Results:**

A factor analysis grouped the 24 items into five factors (life balance/fitness/body image/contact with others/fun). Our qualitative study explained these in more detail and revealed missing aspects. 47.6% of students participated in USP. Potential improvements for USP include program maintenance during the semester break and temporal harmonization with the classes.

**Discussion:**

The qualitative component identified additional reasons for sport activity that were not addressed by the item battery, which provides critical implications for developing item batteries for future research. Our results may help to generate a more target-group-oriented approach to increase physical activity among students, which will reduce sedentary behavior and future disease burden.

## 1. Introduction

 Adolescence and emerging adulthood is a time of physical, social, psychological, and structural changes, which may influence barriers to and motivations for physical activity [1]. University students represent a specific subgroup in this period as they are particularly affected by changing (structural) life circumstances with the start of their studies. Students' daily lives are characterized by sedentary behavior (e.g., attending university classes); however, physical activity in this age group is important, because future patterns of adult health are established already at this stage of life [2]. In addition, obesity resulting from a lack of physical activity in this age group can have adverse health consequences later in life [3].

A variety of studies have been conducted to identify reasons for participating in physical activity and potential barriers to being physically active in adolescence and young adulthood [4–8]. Motivation for physical activity is often measured by asking about its benefits; there is no consistent way of categorizing items, and often an “intrinsic motivation” category has been used [7, 9–11]. The essential benefits identified in former studies have been “health” [6, 11, 12] and “fun” [5, 8, 12].

These former studies were mainly quantitative and followed a similar procedure (i.e., using a predefined, self-reported questionnaire). Results were discussed without reflecting them back to the target group, which is often necessary to gain a deeper insight to the following questions: (1) What are the aspects underlying the individual response categories? (2) What motivates these responses? and (3) Are there other, more specific aspects associated with the response categories that were neglected? So far, these questions have not been answered. By using a mixed-methods approach consisting of a quantitative survey and qualitative reflection, we contribute to a deeper understanding of motivation for physical activity among university students [13].

Moreover, besides gathering information on general motivators for physical activity, further details on sport programs that are offered to university students are also needed. To enhance the benefits of a university sports program, it is important to target the program to the needs of the students. However, there is a lack of target-group-specific recommendations for the future development of university sports, for instance, in Germany. While quantitative results may help to identify how many students participate in university sports, a qualitative analysis is useful in exploring participants' needs and providing concrete suggestions for program improvement.

Consequently, we bridged existing knowledge gaps by using data from a mixed-methods study conducted among university students. We aimed to (1) quantify the importance of predetermined potential reasons for sport activity, (2) reflect the target groups' quantitative results by using qualitative interviews to garner a deeper understanding of the reasons and to identify neglected reasons to analyze content validity, (3) quantify the utilization of the university sports program, and (4) collect information on its potential improvement.

## 2. Materials and Methods

The analyses were based on the Nutrition and Physical Activity Study (NuPhA), a mixed-methods study (explanatory sequential design) including a cross-sectional survey (n=689) and guided face-to-face interviews (n=20) with university students in Germany. We chose this design to gather quantitative information on students' motivations for sport activity and their utilization of the university sports program as a first step. Then, we conducted the qualitative interviews to get a deeper understanding on motivations and utilization as well as to elaborate possible explanations for the quantitative results. The qualitative study part was also used to test the content validity—and therewith the completeness—of the item battery used in the quantitative part. In the past, applying a mixed-methods approach has been shown to be useful since the methods and their results inform each other [14].

This study obtained ethics approval by the Medical Ethics Committee of the Medical Faculty Mannheim, Heidelberg University (2013-634N-MA). All participants provided informed consent before taking part in the study.

### 2.1. Quantitative Study

We conducted an online survey among university students from all over Germany from October 31, 2014, to January 15, 2015. Students were recruited via fliers, mailing lists, social networks, and lecturers. Completing the questionnaire took approximately 30 minutes. Prior to the first question, participants received information on the study aims and data security. Forty gift cards (20 worth €25 and 20 worth €50) were raffled off.

#### 2.1.1. Variables

Motivation: motivation to be physically active was measured via 24 items (e.g.,* I do sports because it relaxes me*), which were answered using five choices (*disagree, tend to disagree, undecided, tend to agree, *and* agree*). The items are based on an established battery by Brown et al. [4]. Since this item battery also included barriers for physical activity, which were analyzed independently in our study following Andajani-Sutjahjo et al. [15], we excluded the items on barriers and added items on motivation from other validated instruments [5–8, 10–12] to cover an even broader range of items. The completeness of the final item battery was tested—as described above—in the qualitative study part (content validity; [14]). Motivation questions were only answered by the students who reported to be physically active (91.9% of the total sample).

Additional individual characteristics: individual characteristics that were included in the analyses were sex, residence change due to start of studies (*yes/no*), and number of semesters studied.

#### 2.1.2. Analyses

First, we performed descriptive analyses of the 24 items on motivation for sport activity and calculated Cronbach's alpha. We conducted a factor analysis with oblimin rotation to summarize the 24 items. All analyses were performed using IBM SPSS Statistics 22 (IBM Corporation, Armonk, USA).

#### 2.1.3. Sample Characteristics

Students (n=689, aged 16–29 years, Table 1) from all over Germany participated in the study (69.5% women; mean age = 22.7 years). 82.0% of the students reported a change in physical activity compared to school. While 36.5% stated that they are more physically active today, 45.4% indicated being less physically active. Most were physically active (91.9%); however, 79.8% stated that they would love to be more physically active (women = 81.8%, men = 75.2%, p = .047).

### 2.2. Qualitative Study

From March till December 2016, 20 interviews were conducted face-to-face by the last author (JH, a female scientist who is trained in interviewing) without the presence of a third person. The students were recruited via fliers and social networks till theoretical saturation was reached. The average duration of the interviews was 41:45 minutes (range = 28–59 minutes). Prior to the first question, participants received information on study aims and data security. All participants received a gift card for participating (worth €20). We used a semistructured interview guide with open-ended questions. Interviews were audiotaped (Olympus DS-2500) and transcribed verbatim.

#### 2.2.1. Analyses

Qualitative content analysis following Mayring [16] was performed to identify themes, patterns, and contradictions by comparing the 20 interviews. Categories were identified based on the quantitative study. Regarding motivation, our analyses revealed five main categories, which were further divided into 18 subcategories. Regarding university sports, we had four main categories. The data were independently coded by two researchers (JH and HD). The comparison revealed high agreement (82%). For coding the data, we used MAXQDA 12 (VERBI Software GmbH; Berlin, Germany).

#### 2.2.2. Sample Characteristics

Students were aged 20–26 years (mean age = 22.8 years). Nine were completing a bachelor's degree (semesters three to six), six were completing a master's degree (semesters two to six), and six were completing a state exam (semesters seven to ten). Eleven participants (55%) stated that they were participating in sports more often compared to their sport activity during school years. Nineteen of the 20 students were physically active; however, 15 said that they would love to do more sports.

## 3. Results

With regard to the quantitative study, the five most critical motives for being physically active were “because it makes me feel good” (completely agree: 73.3%), “because it is healthy” (56.7%), “because it is fun” (55.8%), “to stay fit” (54.1%), and “to achieve balance in daily life” (51.0%). Reliability was good (Cronbach's alpha = 0.853). Factor analysis of the 24 items revealed five factors (*life balance, fitness, body image, contact to others, fun*; KMO=0.871; Bartlett<0.001, Table 2).

In the qualitative study, further information about these five factors and the reasons behind the answers regarding motivations for physical activity have been addressed.* Life balance* included various aspects of “balance in daily life”: an important reason seems to be “to keep a clear head” (S03). This was mentioned in ten of the 20 interviews (S03, S08, S09, S10, S11, S12, S13, S14, S15, and S20). By being physically active, the students try to “reduce stress and calm down” (S08) and “relax a bit and take (their) mind off of everyday problems” (S10). According to them, it feels good to “stop contemplating” (S12), to “switch off” (S13), and to “let the mind wander” (S14). Furthermore, sports are seen as “a source of relaxation” (S20) and a measure to “reduce stress” (S15). Another aspect that was mentioned repeatedly was sports' ability to compensate for the time spent sitting down while studying (S07, S08, and S19).

*Fitness *not only described building endurance (S06, S07, S14, and S19), but also muscle, especially in the back area (S01, S11, and S20). In this context, sports were also mentioned as “preventing back pain” (S18) and counteracting tenseness because of sitting down for extended periods (S01, S18, S19, and S20). Other associations were general health aspects and the need to stay fit overall (S02, S06, S07, S13, and S16).

*Body image* included thoughts on body shape, the wish to be slim, and the feeling of physical well-being after playing sports. Body weight seems to be important to both young men and women. The students do not want to “gain a lot of weight” (S07), which is why they try to “counterbalance any deficits in the diet” (S06) by playing sports. Participants also mentioned that their body metabolism has changed over time, which is why they cannot eat whatever they want now (S07) and need to watch their weight (S18). However, it seems to be important not only to have a slim physique, but also to be athletic and to sculpt one's body (S20). One's personal “body sensation” (S09) seems to be part of the body image as well, which means that participants “feel better” (S30) about themselves after playing sports and if they miss it because there is no time, they do not feel fit and awake (S08).

*Contact with others* described diverse benefits: (a) the development of a feeling of community in sports (S01, S04, S09, S15, S16, and S17), with “group experiences” (S04) as a reason to be physically active, as well as “sharing a sense of achievement with other people” (S15); (b) getting to know new people (S03, S08, S14, and S15) and developing friendships (S08 and S14); (c) spending time together with other people (S06, S12, S14, S15, and S18) and “keeping in touch with friends” (S12); and (d) a positive kind of peer pressure that serves as a motivation to be physically active (S15 and S17).

*Fun* was minimizing barriers to be physically active (S13, S16, and S20). “If it was not fun, (…) then it would definitely be a hassle to go there” (S03). Enjoying physical activity was associated with competition and team sports (S14 and S15) and a sense of self-affirmation seemed to be the source of pleasure for some participants (S06, S17, S19, and S20).

### 3.1. University Sports

In the quantitative study, 47.6% of students stated that they participated in university sports. Because of this, students in the qualitative part of the study were asked about how they would evaluate the program in general, how satisfied they were with it, how often they used it, what may prevent someone from participating, and what could be improved.

The qualitative data showed that, in general, students evaluated their university's sports program positively: “It's fine the way it is. I don't think there's anything that should be changed” (S06). The participants especially liked that many courses were free-of-charge or had very low participation fees (S01, S06, S08, S13, S16, S18, S19, and S20).

The variety of courses available was also seen as an advantage (S10, S11, S14, and S17): “The program offers every kind of activity imaginable, from climbing to kayaking and even field trips” (S14). However, some students expressed mixed feelings: “While there is a wide range of courses available, for me personally, there isn't much that interests me” (S20). Common criticisms were the sizes of the facilities and the number of attendees (S02, S03, S15; S18, and S19): “It's just not a lot fun to share the room with one hundred other people” (S03).

Not all students were taking advantage of the university sports program—some praised the variety of available courses, but still did not attend any of them (S01, S13, S09, and S20). Others, however, gladly took advantage of them (S02, S07, and S17). Participating in the program for the very first time may be difficult for some, as one student explained: “My first experience was rather unpleasant; so, I had to force myself to attend in the beginning. I didn't do very well and was out of breath quickly, which wasn't much fun. But in the end, I started doing more sports because of it” (S07).

The main reasons that prevented students from taking advantage of the program were a lack of time and it not being compatible with their schedules (S07, S08, S10, S11, S14, S16, and S20). Access to the facilities (S04 and S05) seemed to be less of a hindrance.

Possible improvements according to study participants were a broader range of courses available during semester breaks (S03) and further adapting them to the usual lecture times so that students could play sports immediately after finishing their courses for the day (S07). The United States was mentioned as an example of a nation where “sports are an integral part of university life” (S12). In addition, some students expressed the need for more beginner courses to accommodate first-time participants (S19), expanding the sports program (S06), and increasing promotion (S10).

## 4. Discussion

To our knowledge, this study is among the first to combine quantitative and qualitative methods to analyze university students' motivation for sports engagement. Our study underlines the importance of combining quantitative and qualitative research approaches, since our qualitative analysis identified additional reasons for sport participation that were not addressed by the quantitative instrument. However, such a procedure can be helpful to analyze content validity. In addition, we assessed the perception and the utilization of university sports, which is a key form of physical activity in this target group.

The importance of fun, health, and well-being have been stated as key reasons for physical activity in previous studies [5, 6, 8, 11, 12]. Besides analyzing individual items, we used a quantitative factor analysis to group participants' reasons for sports engagement [4]. Our quantitative data revealed 5 factors, which were explained in more detail during our qualitative analysis. For instance, the factor* life balance* was additionally interpreted as “time for recovery,” which was not addressed by the quantitative items. The* fitness* factor included trying to prevent back pain resulting from a sedentary way of life. Concerning the factor* contact with others*, qualitative interviews revealed that playing sports involves a group experience and a shared sense of achievement. Further, students indicated that sports are a source of* fun* and pleasure and can provide self-affirmation. In sum, the qualitative study provided critical suggestions for the further development of quantitative items. Our findings underline the importance of including the target group in the process of developing assessment methods such as questionnaires.

The university sports program was positively evaluated by most of the students. Lack of time was mentioned as the most important barrier for nonparticipation in university sports. This is in line with previous research [12, 15, 17–23]. An aspect that was often criticized by students in our qualitative interviews was overcrowded courses. Students also mentioned that the maintenance of the sports program during semester break and the harmonization of class schedule with the start of sport courses would be beneficial.

### 4.1. Strengths and Limitations

Our study is among the first in combining quantitative and qualitative data und thus comes to profound results. Nonetheless, there are some limitations that should be considered while interpreting our results. First, we used convenience sampling, a type of nonprobability sampling. Therefore, there could be participation bias. Second, participants answered some questions retrospectively; therefore, there may be recall bias. However, this bias was not a problem in our pretests. Third, because of the cross-sectional design of the survey, it is not possible to identify causal relationships. Since our overall aim was to explore motivation and barriers for physical activity, this shortcoming played a trivial role.

## 5. Conclusion

Our study is a lesson in the importance of combining quantitative and qualitative methods. Only if those who are directly affected—in this case, students exposed to sedentary behavior—are involved in the development of survey tools can the reasons for participating in sports be comprehensively and exhaustively assessed. Our findings provide key implications for other researchers working in this field. Nearly half of the students participated in university sports, which underlines their importance. By following the recommendations for improvement made by the students (i.e., maintenance of the sports program during semester breaks and the harmonization of class schedule with the start of sport courses), stakeholders can enable even more students to participate in university sports and to benefit from the program. This would be a crucial step towards an increase in physical activity in this target group and thus a relevant contribution to their current and future health.

## Figures and Tables

**Table 1:** Characteristics of university students participating in the quantitative component of the NuPhA-Study (Germany).

			Male		Female		
	n	%	n	%	n	%	p-value
Social demographics							

Sex	689						
Male	210	30.5					
Female	497	69.5					
Age	689						0.081
up to 20	167	24.2	40	19.0	127	26.5	
21 - 22	170	24.7	48	22.9	122	25.5	
23 - 24	188	27.3	64	30.5	124	25.9	
25 and older	164	23.8	58	27.6	106	22.1	
Mean (SD)	22.69	(2.73)					
Immigrant background	689						0.513
No	593	86.1	178	84.8	415	86.6	
Yes	96	13.9	32	15.2	64	13.4	
Family status	689						0.537
Married	28	4.1	9	4.3	19	4.0	
Committed relationship	360	52.2	103	49.0	257	53.7	
Single	301	43.7	98	46.7	203	42.4	
Residence	689						0.700
Alone	137	19.9	47	22.4	90	18.8	
With a partner	136	19.7	40	19.0	96	20.0	
Shared flat	246	35.7	75	35.7	171	35.7	
Dormitory or elsewhere	170	24.7	48	22.9	122	25.5	
Money per month	678						0.045
Until 550	174	25.7	46	22.2	128	27.2	
551 - 690	139	20.5	34	16.4	105	22.3	
691 - 885	180	26.5	58	28.0	122	25.9	
886 +	185	27.3	69	33.3	116	24.6	

Study-related characteristics							

Study discipline	689						0.018
Social Sciences	86	12.5	20	9.5	66	13.8	
Medicine/Health Care	369	53.6	104	49.5	265	55.3	
Sport Sciences	43	6.3	19	9.0	24	5.0	
Law	46	6.7	21	10.0	25	5.2	
Other disciplines	145	21.0	46	21.9	99	20.7	
Semester	671						0.361
1 - 3	234	34.9	64	31.2	170	36.5	
4 - 5	127	18.9	42	20.5	85	18.2	
6 - 9	187	27.9	55	26.8	132	28.3	
10 +	123	18.3	44	21.5	79	17.0	
Mean (SD)	5.89	(3.51)					

SD = standard deviation.

P-values are based on Chi^2^-tests.

**Table 2:** Factor analysis of 24 items on reasons for being physically active in German university students (NuPhA Study).

**I do sports…**	Factor loadings	Strongly disagree (%)	Disagree (%)	Neither agree or disagree (%)	Agree (%)	Strongly agree (%)	Mean (SD) / Median (IQR)
**Factor 1: Life balance**							
…because it helps reducing anxiety, stress and worries	.823	6.5	7.6	9.9	35.3	40.7	3.95 (1.19) / 4 (1)
…because I need to balance out my everyday life	.791	3.9	4.7	5.5	34.8	51.0	4.23 (1.04) / 5 (1)
…because it distracts me from problems	.721	9.4	18.9	18.9	28.7	24.1	3.39 (1.29) / 4 (2)
…to blow off steam	.700	9.0	14.3	15.2	34.4	27.0	3.57 (1.28) / 4 (2)
…because it relaxes me	.626	3.6	6.6	12.6	40.6	36.5	4.00 (1.09) / 4 (1)

**Factor 2: Fitness **							
…to keep fit	.833	0.8	1.7	2.4	41.0	54.1	4.47 (0.69) / 5 (1)
…because it is healthy	.755	1.1	2.2	5.1	34.9	56.7	4.45 (0.77) / 5 (1)
…to improve my performance	.737	1.4	2.1	6.6	42.2	47.6	4.34 (0.79) / 4 (1)
…because it strengthens my muscles	.687	0.9	3.2	6.6	41.6	47.6	4.33 (0.79) / 4 (1)
…because it makes me feel good	.542	0.6	1.1	2.8	22.2	73.3	4.66 (0.66) / 5 (1)

**Factor 3: Body Image**							
…because it makes me look slim	.855	15.1	14.6	19.5	34.9	16.0	3.20 (1.31) / 4 (2)
…because it helps me to prevent weight gain	.840	17.4	12.3	13.9	37.8	18.6	3.26 (1.38) / 4 (2)
…because I have weight problems	.735	43.9	19.1	13.9	15.0	8.1	2.22 (1.36) / 2 (2)
…because I do something for my physique	.642	5.5	8.2	12.3	41.6	32.3	3.86 (1.13) / 4 (2)
…because I can maintain my sporty appearance afterwards	.541	9.5	13.4	22.6	33.2	21.3	3.43 (1.23) / 4 (1)
….because I look better afterwards	.516	4.3	9.5	17.9	36.6	31.7	3.83 (1.11) / 4 (2)
…because it improves my self-perception	.379	4.1	6.8	16.9	43.3	28.9	3.86 (1.04) / 4 (2)

**Factor 4: Contact to others**							
…because it strengthens my friendships	.879	24.7	21.7	25.5	21.1	7.0	2.64 (1.25) / 3 (2)
...because it helps me maintaining my contacts	.847	26.4	21.4	23.3	21.4	7.6	2.62 (1.29) / 3 (3)
…because sport gives me the possibility to do something in company of others	.812	16.6	19.2	20.9	31.5	11.7	3.01 (1.29) / 3 (2)
…because my friends also exercise	.808	17.8	21.7	19.5	26.0	14.9	2.98 (1.34) / 3 (2)

**Factor 5: Fun**							
…because it is my hobby	.544	3.8	9.2	12.9	26.7	47.5	4.06 (1.15) / 4 (2)
…to measure my strength with others	.541	39.6	24.3	18.3	12.6	5.2	2.20 (1.24) / 2 (2)
…because it is fun	.491	1.0	4.8	7.9	30.6	55.8	4.37 (0.88) / 5 (1)

Factor analysis with oblimin rotation; Kaiser-Meyer-Olkin=0.871, Bartlett<0.001; n=602; only students being physically active answered the question on the reasons.

SD = standard deviation; IQR = interquartile range.

## Data Availability

The data analyzed during the current study are available from the corresponding author on reasonable request.
